# Testing the Role of Dorsal Premotor Cortex in Auditory-Motor Association Learning Using Transcranical Magnetic Stimulation (TMS)

**DOI:** 10.1371/journal.pone.0163380

**Published:** 2016-09-29

**Authors:** Carlotta Lega, Marianne A. Stephan, Robert J. Zatorre, Virginia Penhune

**Affiliations:** 1 Department of Psychology, University of Milano-Bicocca, Milano, Italy; 2 Brain Mind Institute, Ecole Polytechnique Fédérale de Lausanne, Lausanne, Switzerland; 3 Department of Psychology, Concordia University, Montreal, QC, Canada; 4 Montreal Neurological Institute, McGill University, Montreal, QC, Canada; 5 International Laboratory for Brain, Music and Sound Research (BRAMS), University of Montreal, Montreal, QC, Canada; Northwestern University, UNITED STATES

## Abstract

Interactions between the auditory and the motor systems are critical in music as well as in other domains, such as speech. The premotor cortex, specifically the dorsal premotor cortex (dPMC), seems to play a key role in auditory-motor integration, and in mapping the association between a sound and the movement used to produce it. In the present studies we tested the causal role of the dPMC in learning and applying auditory-motor associations using 1 Hz repetitive Transcranical Magnetic Stimulation (rTMS). In this paradigm, non-musicians learn a set of auditory-motor associations through melody training in two contexts: first when the sound to key-press mapping was in a conventional sequential order (low to high tones mapped onto keys from left to right), and then when it was in a novel scrambled order. Participant’s ability to match the four pitches to four computer keys was tested before and after the training. In both experiments, the group that received 1 Hz rTMS over the dPMC showed no significant improvement on the pitch-matching task following training, whereas the control group (who received rTMS to visual cortex) did. Moreover, in Experiment 2 where the pitch-key mapping was novel, rTMS over the dPMC also interfered with learning. These findings suggest that rTMS over dPMC disturbs the formation of auditory-motor associations, especially when the association is novel and must be learned rather explicitly. The present results contribute to a better understanding of the role of dPMC in auditory-motor integration, suggesting a critical role of dPMC in learning the link between an action and its associated sound.

## Introduction

Auditory-motor integration is crucial for the learning and production of music and speech. Based on work in both animals and humans, the network of brain regions engaged in linking sound and action is thought to involve the auditory dorsal stream, including the posterior auditory, inferior parietal and premotor cortices [1–6]. Hickok and Poeppel [6] proposed a dual–stream model specific for speech processing, in which the ventral auditory stream is responsible for mapping sounds onto meaning and the dorsal stream is critical for mapping sounds onto representations of articulatory movements. Lately, Warren et al. [7] proposed a general model for auditory-motor transformations and pointed out the critical role of the dorsal auditory pathway, where the planum temporale analyzes sounds that are relevant for the motor system, such as speech and melodies, which are then transformed into motor representations in prefrontal, premotor and motor regions.

The premotor cortex (PMC) has traditionally been divided into ventral (vPMC) and dorsal (dPMC) subregions, both of which represent a crucial node in the auditory-motor network. In the visual domain it has been proposed that these regions are involved in direct and indirect visuo-motor associations, respectively [8–10]. Direct associations are those that encode a fixed, one-to-one mapping between an object and an action, for example a cup and the hand shape needed to grasp it. In contrast, indirect sensorimotor associations are more abstract and flexible and, once established, a sensory stimulus may represent a conditional rule indicating which response to select among different possible responses [3,9,11,12]. In parallel with the visual system, previous neuroimaging studies [1,2,13] suggest a similar functional dissociation in the auditory domain. In particular, the dPMC is hypothesized to be important for learning flexible and/or arbitrary associations between sounds and actions [1,3,13] and for selecting movements in the appropriate context [10]. Consistent with its role in abstract and higher-order aspects of sensorimotor integration [3,8,11,14], activity in the dPMC has been shown to be sensitive to a rhythm’s metric structure [15], and inactivation of the dPMC impairs conditional motor behaviours [16]

In the domain of music, studies in both trained and untrained individuals show that dPMC is engaged when people listen to, learn, play or imagine musical sequences. In all of these cases, individuals are learning, or have an established association between sound and action. For example, in musicians dPMC is engaged when either listening to music without playing or when playing without auditory feedback [17,18]. Further, dPMC is engaged when musicians listen to and learn to play new melodies [5]. Relatively few studies have investigated the specific brain networks involved in learning new auditory-motor associations. In an early study, Bermudez and Zatorre [19] showed increased activity in rostral dPMC after non-musicians had been trained to associate four chords to four button presses. Similarly, Lahav et al., [20] demonstrated the activation of the PMC when subjects listened to melodies that they had been trained to play, but not when they listened to un-trained melodies composed of different notes. This result is in line with previous studies in musicians showing premotor activation when they listened to a rehearsed musical piece [17,21]. In a recent fMRI study, non-musicians were trained to play short melodies on the piano. After training, dPMC showed greater activation when people listened to or imagined playing the melodies they had learned [13]. Similarly, a recent study in non-musicians showed that dPMC was engaged during learning to play short piano melodies, and that the degree of engagement was related to improvements in performance on a pitch matching task [2].

Despite strong correlational evidence from the studies reviewed above, there is no direct causal evidence that dPMC is required for learning new auditory-motor associations. Previous studies using inhibitory rTMS to disrupt dPMC function have shown that that it perturbs auditory-motor synchronization [22,23], but its effect on auditory-motor learning has not been examined. Therefore, in the current study we used rTMS to disrupt dPMC function as non-musicians learned to associate a musical note with a key press. To test the effect of rTMS on auditory-motor learning, we used a variation of the paradigm developed by Chen and colleagues [2] in which non-musicians learn a set of auditory-motor associations through melody training. Further, we tested the role of dPMC in learning a new auditory-motor association in two contexts: first when the sound to key-press mapping was conventional and ordered (low to high mapped on to left to right key order), and then when it was scrambled. In the first context, when the mapping is conventional, even non-musicians may have pre-existing associations between pitch and spatial location. Indeed, previous studies demonstrate that non musicians tend to associate sounds in an ascending musical line with a spatial mapping from left to right [24,25], and that this conventional, ordered mapping facilitates action planning and sequence learning [26–28]. In the second context, the mapping between pitch and key location was scrambled, guaranteeing that participants would have to learn an entirely new set of arbitrary auditory-motor associations. We hypothesized that if the dPMC is crucial in learning new auditory-motor association, then rTMS over dPMC should interfere with that learning, and that interference would be greater in the scrambled compared to the conventional pitch-to-key mapping.

## Materials and Methods

### Participants

Fifty young, healthy participants took part in these experiments. Twenty-four were tested in Experiment 1 (7 M; mean age = 22.37; SD = 3.80) and twenty-six in Experiment 2 (10 M; mean age = 22.88; SD = 3.68). In each experiment half of the participants (12 in Exp 1 and 13 in Exp 2) were randomly assigned to the dPMC stimulation and the other half to the V1 stimulation. Participants were selected to have little musical training (Experiment 1: mean years of musical training = 0.58; SD = 0.77; Experiment 2: mean years of musical training = 0.92; SD = 1.01). They were all right-handed, according to the Edinburgh Handedness Inventory [29]. Prior to the experiments, each participant filled out a questionnaire to assess whether it was safe for them to undergo TMS. None of the volunteers reported neurological or psychiatric problems, seizures, or was taking any medication that could interfere with neuronal excitability. All participants provided written informed consent. The local ethics committee (Comité d’éthique de la recherche en santé (CERES)) approved the protocol, and participants were treated in accordance with the Declaration of Helsinki.

### Procedure, tasks and stimuli

**Fig 1:**
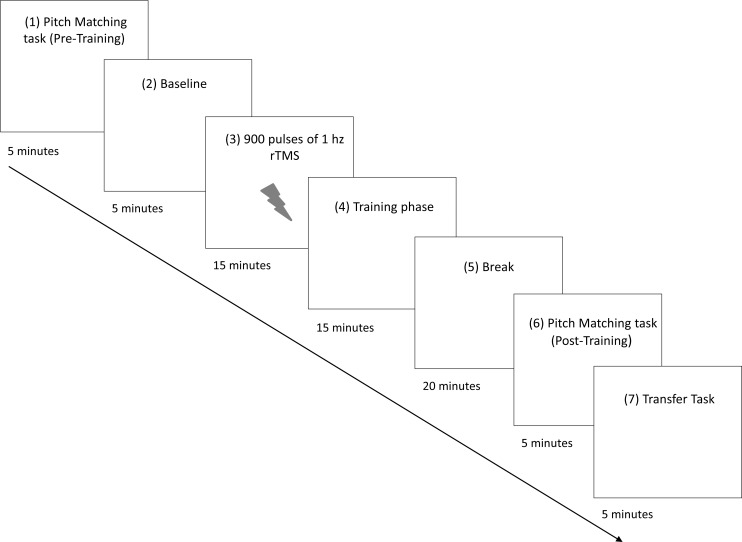
The timeline of the Experiment 1 and 2. Participants were asked to perform the Pitch-to-key press matching task (1) and the Baseline (2) task before the TMS stimulation. After that, 1 Hz rTMS was applied over the dorsal premotor cortex (dPMC) and over the primary visual cortex (V1) (3) before the 3 Blocks of training (4). At least 40 minutes after the end of the stimulation (5) participants performed again the pitch-to-key press matching task (6) and the Transfer task (7).

The paradigm used for Experiments 1 and 2 was based on that developed by Chen and colleagues [2]. The timeline for both experiments is shown in Fig 1. At the beginning of each experiment, participants were tested on a pitch-to-keypress matching task (referred to as “Pitch Matching”) to assess their ability to associate each of four pitches (C, D, E, G) with one of four keys on the computer keyboard. Participants heard single pitches and had to match them to the keys using the four fingers of the right hand (not including the thumb). The letters on the keys were covered so participants had to associate each pitch with a location or keypress, not with the letter on the key. Each of the four pitches was presented 10 times for a total of 40 trials. Trials were pseudo-randomly ordered (to avoid the same pitch occurring twice in a row), and no auditory feedback was given, in order to rule out possible learning effects [2,20]. Key-press responses and reaction times were recorded by the computer.

**Fig 2:**
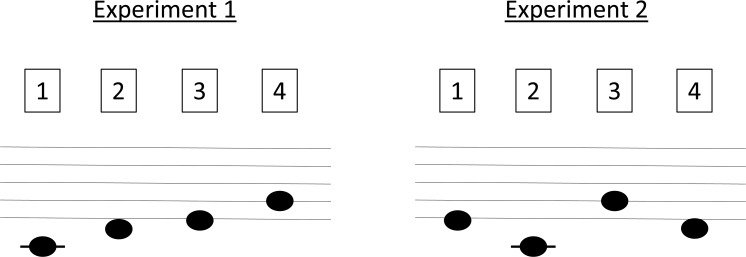
The auditory-motor mapping of Experiment 1 and Experiment 2. In Experiment 1 tones were ordered from low to high and corresponded to the spatial position from left to right on the computer keyboard (C = key 1, D = key 2, E = key 3, G = key 4). In Experiment 2 the order of notes was scrambled (E = key 1, C = key 2, G = key 3, D = key 4).

In Experiment 1 pitches were mapped to keys in a conventional, ordered low-to-high/left-to-right mapping: C = key 1, D = key 2, E = key 3, G = key 4. In Experiment 2 the mapping of pitches to keys was scrambled: E = key 1, C = key 2, G = key 3, D = key 4 (See Fig 2).

Participants were then trained on the pitch-to-key association through a melody playback task in which the same four pitches were used. The first block of training occurred immediately before application of the rTMS and served as a baseline (Pre-training). Participants then received 900 pulses of inhibitory 1 Hz rTMS over either the dPMC or over the control site (primary visual cortex, V1). For details of the rTMS stimulation see below. Participants then performed three additional blocks of melody playback (Training).

On each trial of training participants first listened to a melody and were then asked to play it back using the four keys on the computer keyboard. Each key press evoked a specific tone. Thus, participants heard both the target and their own responses, which was designed to allow them to learn the key-to-pitch associations. Melodies were five-note sequences in which the same four notes were rearranged to create different sequences. In each melody, three pitches (C, D, G) were used once and a fourth (E) was used twice, (e.g., D E C E G). There were 45 different melodies in total, and each block of training contained 15 melodies. Each Block lasted approximately 5 minutes, for a total of 15 minutes of training.

Following the three blocks of training, participants were retested on the Pitch Matching task (Post-training). The inhibitory effect of 1 Hz rTMS is usually thought to last for a maximum of 20–30 minutes [30]. To be sure that the effect had dissipated before retesting on the Pitch Matching task, we allowed 40 minutes to elapse. To do this we introduced a short break after the end of the last training block. This break was on average 20 minutes and participants were simply asked to relax, and not to listen to music, or type on a computer keyboard.

Finally, in order to assess the flexibility of the pitch-to-key associations, we tested participants on one block of transfer in which they listened to and reproduced a single novel melody repeated 15 times (Transfer).

All pitches and melodies were presented through headphones, and were created with the "GarageBand" music editing software (GarageBand 6.0.4, Apple Inc. 2011) using a synthesized piano timbre. Each of the four tones lasted 600 msec. Keypress responses and RTs (Reaction Times) were recorded by the computer.

### Transcranical magnetic stimulation

**Fig 3:**
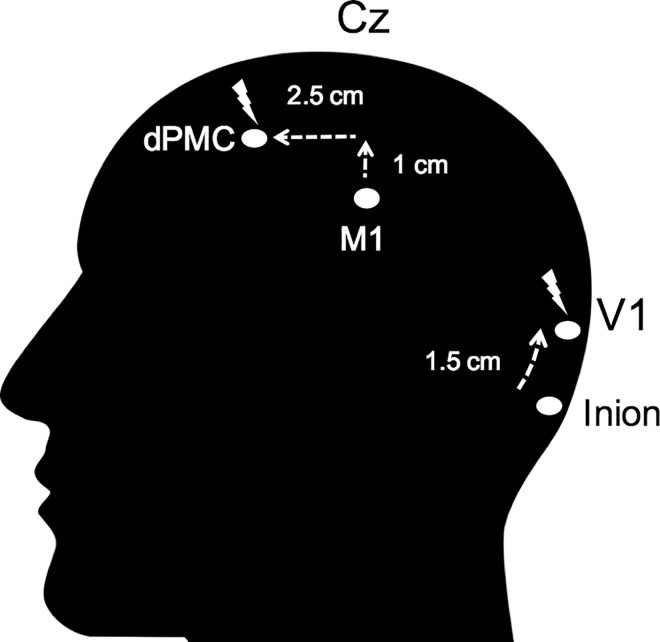
Diagram of the stimulated site. The dPMC was located 1 cm medial and 2.5 cm anterior at the same laterality as the motor ‘hot-spot’ (M1), defined as the site where the largest MEPs could be evoked in the relaxed first dorsal interosseus (FDI) muscle. The site for V1 stimulation was localized as the point lying 1.5 cm superior to the inion on the midline.

Each participant underwent inhibitory 1 Hz TMS stimulation over the dPMC or over V1. Stimulation intensity was 90% of individual active motor threshold (aMT). The mean stimulation intensity over dPMC was 38.60% of maximum stimulator output and 41.28% over V1, with no significant difference between the two areas (*p* = .15). For each of the sites, 900 pulses were applied at a frequency of 1 Hz (train duration 15 min). The site for dPMC stimulation was located 1 cm medial and 2.5 cm anterior at the same laterality as the motor ‘hot-spot’ [31] defined as the site where the largest MEPs could be evoked in the relaxed first dorsal interosseus (FDI) muscle (see Fig 3). The site for V1 stimulation was localized as the point lying 1.5 cm superior to the inion on the midline [32–34]. We checked in each participant whether stimulation over the defined dPMC evoked any MEPs and moved the coil 0.5 cm anterior in four subjects where this was the case [35]. TMS was applied through a 70 mm figure-of-eight coil, using a Super Rapid Biphasic Stimulator (Magstim, Whitland, UK) with the handle pointing 45° postero-laterally away from the midline for both M1 and the dPMC. For the V1 control site the coil was placed with the handle pointing upward, parallel to the inion-nasion line. A TMS neuronavigation system (Brainsight, Rogue Research Inc., Canada) was used to ensure a constant coil position during the 15 minutes of stimulation. The aMT was determined according to standard procedure during slight tonic contraction of the FDI muscle (20% of maximal force), using the software based ‘adaptive method’ developed by Awiszus [36] (Motor Threshold Assessment Tool (MTAT, version 2.0: http://www.clinicalresearcher.org/software). An MEP ≥ 200 μV peak-to-peak amplitude was fed back to the software as valid response. EMG recordings were obtained from the right FDI muscle, with conventional surface electrodes in a belly-tendon montage. Signals were amplified, bandpass filtered (1 Hz– 2 kHz) and sampled at a rate of 10 kHz.

## Results

Analysis were performed on both reaction times and accuracy. No significant effect of TMS was found for the reaction times. Thus, we report here only the analysis on the accuracy scores.

### Experiment 1

#### Pitch Matching Task

**Fig 4:**
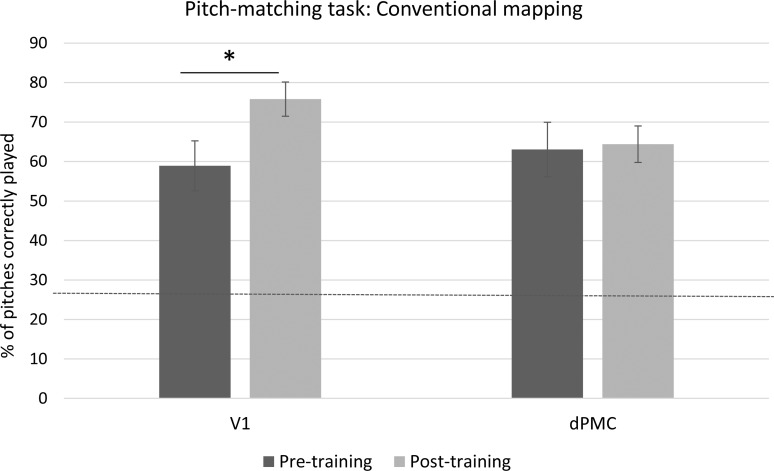
Results of the Pitch matching task of Experiment 1 (ordered mapping). Columns represent average data with standard error bars. The y-axis represents percent correct scores plotted across the TMS site (dPMC and V1). As indicated by the asterisk, TMS over the dPMC significantly reduced participants’ accuracy compared to the V1 stimulation. Horizontal dashed line indicates the level of chance (25%).

A 2x2 repeated-measures ANOVA with Session (Pre- and post- melody playback training) as the within-subjects variable and TMS location (dPMC and V1) as the between-subjects variable was carried out on the percentage of correctly played pitches. The ANOVA revealed a significant interaction between Session and TMS location (F(1,22) = 6.08, *p* = .02, η_p_^2^ = .21) (see Fig 4). Post-hoc comparisons (Bonferroni-Holmes correction) revealed that the V1 group showed a significant improvement in pitch matching performance between the first and the second session, (*t*(11) = 3.55, *p* = .02), but the dPMC group did not (*t*(11) = .32, *p* = .75). Importantly, there were also no significant differences between the V1 and dPMC groups at pre-test (*p* = .66).

#### Training

**Fig 5:**
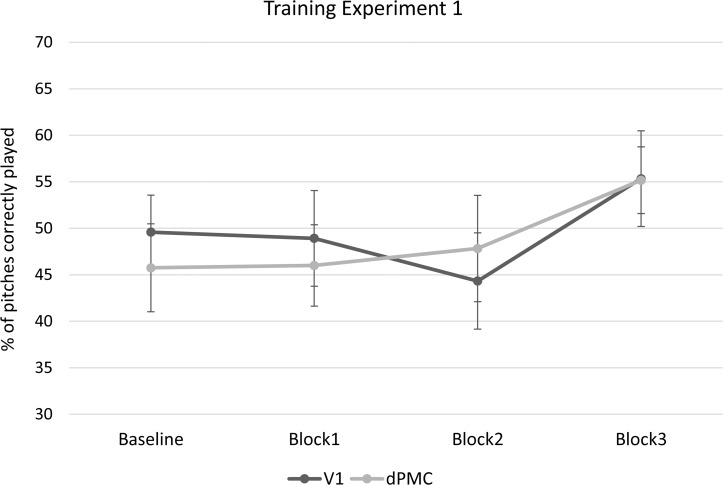
Results of the training phase of the study of Experiment 1. Graph represents the percentage of pitches correctly played plotted across blocks of trials (Baseline, Block 1, Block 2 and Block 3) for the dPMC and the V1 groups. Error bars represent ±1 SEM.

A 4x2 repeated-measures ANOVA with Session (Baseline, Block 1, Block 2, Block 3) as the within-subjects variable and TMS location (dPMC and V1) as the between-subjects variable was carried out on the percentage of correctly played pitches. The analysis revealed a significant main effect of Session (F(3,66) = 5.25, *p* < .01, η_p_^2^ = .19). Neither the main effect of TMS (*p* = .88), nor the interaction between Session and TMS (*p* > .48) reached significance. Post-hoc comparisons with Bonferroni correction showed that for both groups performance improved significantly between Blocks 1 and 3 (*p* = .05) and Blocks 2 and 3 (*p* = .002) (see Fig 5). Since we expected a linear improvement across the four Blocks we also conducted a linear trend analysis, which revealed a significant linear effect of Session (F(1,22) = 4.69, *p* = .04, η_p_^2^ = .18), but no interaction between Session and TMS location (F(1,22) = .78, *p* = .39, η_p_^2^ = .03).

#### Transfer task

To test the effect of TMS on transfer to learning a new melody, we compared the percentage of correctly played pitches on the Transfer block between the V1 and the dPMC group using an independent samples t-test. The analysis showed no significant difference between the two groups (*t*(22) = .18, *p* = .86). In addition, we used a second measure of learning, the number of 100%-correct trials. This analysis also showed no significant difference between groups (*t*(22) = .00, *p* = 1.0).

### Experiment 2

#### Pitch Matching Task

**Fig 6:**
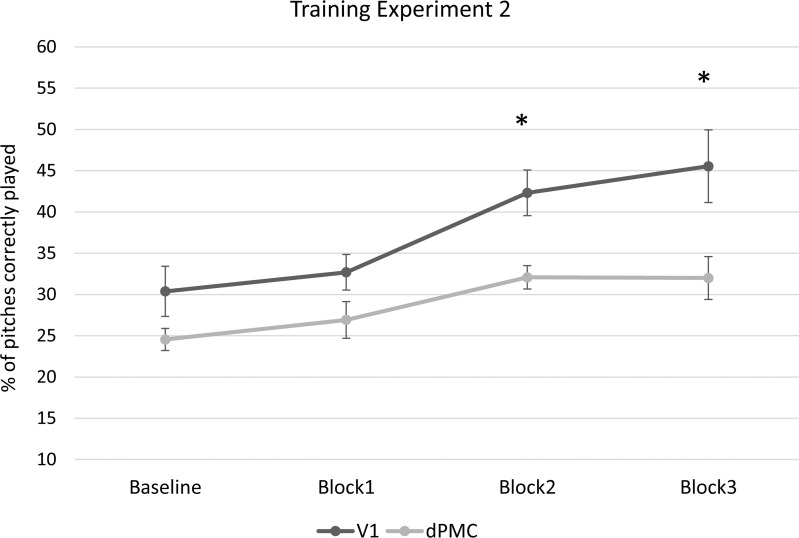
Results of the Pitch matching task of Experiment 2 (scrambled mapping). Columns represent average data with standard error bars. The y-axis represents percent correct scores plotted across the TMS site (dPMC and V1). As indicated by the asterisk, TMS over the dPMC significantly reduced participants’ accuracy compared to the V1 stimulation. Horizontal dashed line indicates the level of chance (25%).

The outcome of this task was overall much more difficult than Experiment 1, as expected. This is confirmed by comparing the pre-training performance of both groups in Experiment 1 and Experiment 2, showing a significance difference between the two mappings, (*t*(48) = 6.04, *p* < .01). Moreover, in Experiment 2 performance pre-training did not differ from chance for either the premotor group (*t*(12) < 1, *p* = .56) and the V1 group (*t*(12) < 1, *p* = .66), whereas it did in Experiment 1 (premotor group (*t*(11) = 5.51, *p* < .001); V1 group (*t*(11) = 5.37, *p* < .001)). A 2x2 repeated-measures ANOVA with Session (Session 1, before training and Session 2, after training) as the within-subjects variable and TMS location (dPMC and V1) as between-subjects variable was carried out on the percentage of correctly played pitches. The ANOVA revealed a significant interaction between Session and TMS location (F(1,22) = 4.98, *p* = .03, η_p_^2^ = .17). Post-hoc comparisons (Bonferroni-Holmes correction) revealed a significant improvement between the first and the second session for the V1 group (*t*(12) = 4.55, *p* = .004), but not for the dPMC group (*t*(12) = 1.84, *p* = .16). Importantly, there were no differences between groups at Pre-test (*t*(24) = .19, *p* = .85), and the V1 group out-performed the dPMC group at Post-test (*t*(24) = 2.87, *p* = .02) (see Fig 6).

#### Training

**Fig 7:**
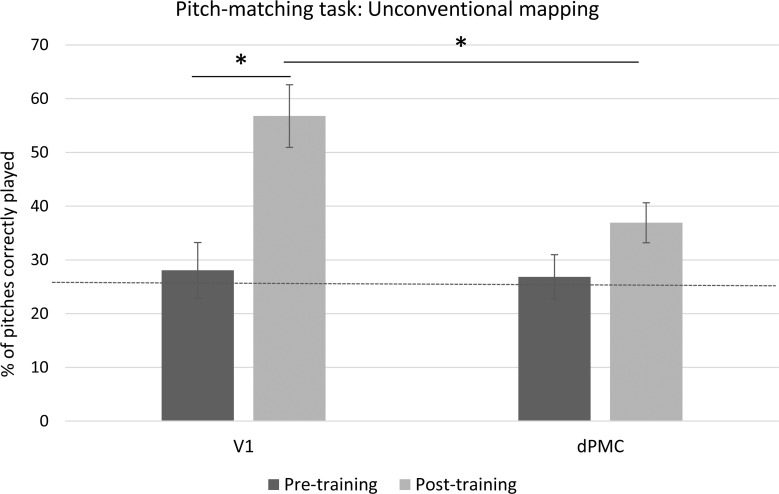
Results of the training phase of the study of Experiment 2. Graph represents the percentage of pitches correctly played plotted across blocks of trials (Baseline, Block 1, Block 2 and Block 3) for the dPMC and the V1 groups. As indicated by asterisks, TMS over the dPMC significantly reduced participants’ accuracy in Block 2 and 3 compared to the V1 stimulation. Error bars represent ±1 SEM.

A 4x2 repeated-measures ANOVA with Session (Baseline, Block 1, Block 2, Block 3) as within-subjects variable and TMS (dPMC and V1) as between-subjects variable was carried out on the percentage of pitch correctly played. Analysis revealed a significant main effect of Session, (F(3,72) = 18.30, *p* < .01, η_p_^2^ = .43). Post-hoc comparisons (Bonferroni-Holmes correction) showed that both the Baseline and the Block 1 significantly differed when compared to both the Block 2 and Block 3 (*p* < .001). The main effect of TMS was also significant (F(1,24) = 8.58, *p* < .01, η_p_^2^ = .26), indicating significantly higher scores for the V1 compared to the dPMC group. Particularly, post-hoc comparisons (Bonferroni-Holmes correction) revealed that the V1 group and the dPMC group differed significantly for Block 2 (*t*(24) = 3.29, *p* = .01) and Block 3 (*t*(24) = 2.64, *p* = .04). The groups did not differ at Baseline or Block 1 (p = .09) (see Fig 7). There was no significant interaction between Session and TMS location (F(3,72) = 2.12, *p* = .10, η_p_^2^ = .08).

Using a linear trend analysis confirmed a significant linear effect of Session (F(1,24) = 39.15, *p* < .01, η_p_^2^ = .62), but also revealed a significant interaction between Session and TMS location (F(1,24) = 4.35, *p* = .04, η_p_^2^ = .15), indicating that the learning rate differed across groups. Specifically, this was due to a stronger linear effect for the V1 group (*p* < .001), compared to the dPMC group (*p* =. 01) (See also Fig 7). Indeed, considering the two groups separately, post hoc analysis (Bonferroni correction) revealed that the V1 group showed a significant improvement between the baseline and Block 2 (*p* < .001) and Block 3 (*p* = .002) and between Block 1 and both the Block 2 (*p* = .01) and Block 3 (*p* = .01). Conversely, the dPMC group only showed a significant improvement when comparing Baseline to Block 2 (*p* = .03).

#### Transfer task

The transfer task was analysed using an independent t-test on the percentage of pitches correctly played between the V1 and the dPMC group. Analysis showed no significant difference between the two groups (*t*(24) = 1.39, *p* = .17). Notably, when we considered the number of 100% correct melodies, the analysis revealed a near significant difference between the two groups (*t*(24) = 1.93, *p* = .06), with the V1 group out-performing the dPMC group.

## Discussion

The current results provide some of the first direct causal evidence in humans that dPMC is involved in the learning and expression of auditory-motor associations. Inhibitory 1 Hz rTMS over dPMC impaired participants’ ability to learn the association between a pitch and a key-press in two independent samples, and this effect was greatest when they were required to learn an unconventional, novel association. These findings are consistent with the hypothesized role of the dPMC in encoding sensory-motor associations, particularly when they are complex or abstract. It is also consistent with findings of previous neuroimaging studies showing that the dPMC is part of a network of regions engaged during learning of auditory-motor associations in the context of music. Finally, our finding that learning of the conventional low-to-high/left-to-right mapping was less impaired by rTMS indicates that some auditory-motor associations may be learned implicitly in the absence of explicit musical training.

Evidence from animals and humans suggests that dPMC is important for learning and expression of abstract or higher-order sensory-motor associations [8,11,14,37]. Globally, evidence from electrophysiological studies in animals has shown that neurons in the PMC respond to auditory and visual stimuli that are linked to known actions [38]. As described in the Introduction, the premotor cortex can be subdivided into dorsal and ventral subregions [9,39], both of which are part of the dorsal auditory stream that links auditory and motor representations. These regions have been found to be active in both musical and speech contexts, when interactions between auditory and motor systems are critical [3,7,40]. In the visual domain it has been proposed that the vPMC and dPMC are involved in direct and indirect visuo-motor transformations, respectively [8,9]. Specifically, vPMC seems to be critical anytime there is a direct mapping from sensory information into the motor system [3,9]. In the grasping movement, vPMC neurons are responsible of processing the shape of an object, and selective lesions of the vPMC in the macaque monkey impair hand shaping, leaving sensory processing undamaged [41]. In contrast, the dPMC is thought to be involved in indirect or higher-order sensorimotor integration [3,9] including the selection of movements that are conditionally linked to a sensory stimulus [11,12,42–44]. This conclusion comes largely from studies in the visual system, where it has been demonstrated in monkeys that the inactivation of the dPMC, but not of the vPMC, impairs conditional motor behaviours [16]. Taken together, there is compelling evidence that both the learning and the performance of arbitrary sensorimotor mappings in conditional associative tasks depends on a complex neural network that includes the dPMC [12,41,44–48]. This functional property of the dPMC perfectly fits with the results of the Pitch matching task in both Experiment 1 and Experiment 2, where a conditional motor response driven by a sensory input is required because participants must decide which movement to select among four competing alternatives.

Interestingly, we also observed that rTMS over dPMC reduced the expression of learning at recall, and slowed learning when the auditory-to-motor mapping was novel, but not when it was conventional. The finding that learning was impaired for the novel (unconventional) mapping only, appears to be consistent with the role of the dPMC in learning new associations, especially when those associations are more abstract and/or complex. Indeed, the manipulation of the pitch-key mapping in the second experiment allowed us to test the hypothesis that dPMC engagement would be more important when learning a more complex or unfamiliar auditory-motor association: scrambling the natural pitch-to-key mapping forced participants to explicitly learn the associations rather than relying on a pre-existing mapping from low to high. Previous studies in both the visual [12] and the auditory [49] domain reported a correlation between activation of the dPMC and complexity of the task. Amiez et al. [12], analysing the brain activation during the learning and the execution of conditional visuo-motor responses, reported that the dPMC is the only area modulated by the number of visuo-motor mappings to be acquired, i.e. the dPMC is sensitive to the difficulty of the task. Combined with these findings, our results show that dPMC is critical in learning conditional sensorimotor associations in the auditory, as well as the visual modality. Thus, we cannot exclude that the greater interference of the TMS observed in Experiment 2 is correlated with the greater involvement of the dPMC once an unconventional (more difficult) mapping is required. The results of the transfer task seem to go in the same direction: when we considered the number of 100% correct melodies, results revealed a trend for the control group to perform better compared to the premotor group in Experiment 2, but not in Experiment 1. These results of the transfer task partially support the role of the dPMC in acquiring new auditory-motor associations. Notably, the main difference between the transfer task and the training blocks lies in the fact that during training the sequences were novel for every trial, whereas in the transfer task the auditory-motor sequence was always the same. Based on the concept that vPMC is more important for direct associations, we could also hypothesize that inhibiting this region via rTMS could have a greater impact on the transfer task, compared to the dPMC stimulation.

Another interesting result of these experiments is that rTMS interfered with learning of the novel, but not the standard left-to-right/low-to-high mapping. This is consistent with evidence showing that some musical associations can be learned without explicit training, supporting the idea that some abilities can be acquired just by being exposed to music [50]. Our results are also consistent with studies that demonstrated the so-called SMARC effect (Spatial Musical Association of Response Codes). They showed that higher pitches facilitate (in terms of speed and accuracy) up\right motor responses, and low pitches facilitate down\left motor responses, even when tones were irrelevant to the task [24,25,51]. The SMARC effect would explain the fact that pitch-mapping performance for Exp I was above chance even before training, but for Exp 2 it was not. Moreover, hearing tones with a conventional mapping facilitates sequence learning in the serial reaction-time task [26,27]. Similarly, Keller and Koch (28) demonstrated faster action planning when the mapping between keys and tones was compatible than when it was incompatible.

We have proposed that rTMS interfered with learning of the key-to-tone mapping in Exp 2 because it was unconventional and novel. In addition, the key-to-tone mapping was not ordered in a spatially sequential manner, i.e., adjacent keys did not correspond to adjacent tones. Thus, future experiments could test an unconventional mapping that is still sequentially ordered, i.e. left-to-right, high-to-low. Further, it is also possible that based on the functional dissociation between dorsal and ventral premotor cortex, the conventional low-to-high mapping may be encoded in the vPMC because more direct and implicit, rather than in the dPMC. Thus future studies could compare the effects of rTMS over both ventral and dorsal regions.

Dorsal premotor cortex is part of a network of regions previously shown in neuroimaging studies to be engaged during learning of auditory motor associations in the context of music [3,13,19,20]. In particular, the study on which our paradigm is based demonstrated that left dPMC was engaged during melody learning, and that activity in this region was related to performance [2]. The left dPMC was chosen as site of interest based on previous studies on auditory-motor learning, which identified more significant changes in the left as compared to the right dPMC after auditory-motor training (2, 13). In the future, it would be interesting to compare the effects of left and right dorsal premotor cortex stimulation. The role of the dPMC in auditory-motor integration is likely based on its pattern of connectivity. Indeed, neuroanatomical studies in non-human primates show that the dorsal and ventral PMC are directly connected to both the posterior temporal gyrus and the primary motor cortex (M1), which makes them a critical node in connecting and integrating auditory and motor information [1–3,5,13,52]. Human studies using diffusion tensor imaging (DTI) to assess language pathways indicate that there are similar connections between the Superior Temporal Gyrus (STG) and the PMC via a dorsal route along the arcuate and superior longitudinal fasciculi, although there is controversy about the precise organization of these fibers [53–56]. Nonetheless, premotor cortices are one link in a complex network of brain regions, which includes cerebellum, posterior auditory and inferior parietal cortices. Studies in both animals and humans have already demonstrated the involvement of parietal, sensorimotor and premotor cortices in the control of movement when the integration of spatial, sensory and motor information is required [57,58]. By analogy with the functional division between ventral and dorsal stream proposed in the visual system, different models suggested that the dorsal auditory stream would be responsible for preparing motor responses from incoming auditory information and in the localization of sounds in space [7,40,59,60]. This concept of the dorsal stream not only unifies its function between vision and audition, but also theorizes its role in auditory-motor integration, critical for both music and speech.

Finally, previous work shows that the rTMS protocol used in this experiment is effective in interfering with cortical excitability over the dPMC [22,61]. Moreover, localizing the stimulation site relative to the M1 hot-spot (2.5 cm anterior and 1 cm medial to M1) [31], allowed us to take into account inter-individual differences in the functional architecture of the brain. However, this approach may not provide the same accuracy and precision as fMRI–guided stimulation [62–64]. In the future, the use of fMRI-guided TMS localization could increase stimulation precision, reducing the variability across subjects, and potentially strengthen some of the current results.

In sum, this study is the first to demonstrate a causal role for the dPMC in learning and implementation of auditory-motor associations. Our findings show that inhibitory rTMS over dPMC impairs the ability to learn and apply auditory-motor associations, and that this effect is greater when a novel association must be explicitly acquired. The present results contribute to a better understanding of the role of dPMC in auditory-motor integration, suggesting its critical role in learning the mapping between an action and its associated sound, a key function allowing us to speak and to play music.

## Supporting Information

S1 File Data set of Experiment 1 and Experiment 2. The Group variable represents the four experimental groups. Mapping variable: 1 = conventional mapping (Experiment 1), 2 = unconventional mapping (Experiment 2); TMS variable: 1 = V1 (primary visual cortex stimulation), 2 = dPMC (dorsal premotor cortex stimulation); Dependent variables calculated as the percentage of correctly played pitches: pitch matching task pre and post training, the baseline, the 3 blocks of training and the transfer task. The baseline, the 3 blocks of training and the transfer task are also reported as number of 100% correct melodies.
(CSV)
